# Background uncertainty does not increase risk aversion in decision making

**DOI:** 10.1038/s41598-024-73650-y

**Published:** 2024-10-29

**Authors:** Johannes Leder, Philipp Chapkovski, Astrid Schütz, Thomas Lauer, Özgür Gürerk

**Affiliations:** 1 Department of Psychology, University of Bamberg, 96045 Bamberg, Germany; 2 Faculty of Social Sciences, University of Duisburg-Essen, 47057 Duisburg, Germany; 3 Erfurt Laboratory for Empirical Research, University of Erfurt, 99089 Erfurt, Germany; 4 Faculty of Management, Economics and Social Sciences, University of Cologne, 50923 Cologne, Germany

**Keywords:** Background uncertainty, Risk-taking behavior, Ambiguity, Risk, Uncertainty, Decision making, Human behaviour, Risk factors

## Abstract

Some theories in economics and psychology propose that *background uncertainty,* which is uncertainty that is independent of a person’s actual decision, can alter people’s risk-taking behavior with respect to that decision. However, previous empirical research mostly relying on single experiments is inconclusive regarding the existence of this effect. Here, we systematically investigate the effect of background uncertainty on decision-making. After reviewing the literature, we argue that two types of background uncertainty should be distinguished: (a) *background ambiguity*, where the decision maker does not know the probability of the outcomes of the background event, and (b) *background risk*, where the outcome probabilities are known. We tested the hypotheses (i) that background uncertainty does affect risk-taking in the decision at hand, and (ii) the type of background uncertainty moderates that effect. In four experiments (total *N* =863), we induced background uncertainty (ambiguity or risk) using different methods and measured risk-taking with multiple behavioral tasks. We did not find a significant effect of background uncertainty on risk-taking behavior.

## Introduction

People frequently make decisions under uncertainty. In some situations, uncertainty is clearly attributable to the decision at hand, while in other situations there may be other sources of uncertainty in the background, like the possibility of the eruption of a nearby volcano. Hence, background uncertainty is a contextual factor that is independent of the decision itself but is likely to influence the decision. Yet researchers of decision-making typically focus only on uncertainty that is inherent to a given decision, i.e., uncertainty in the foreground. Whereas the decision maker can avoid such uncertainty by choosing the safe option, *background uncertainty* refers to uncertainty that the decision maker cannot influence.

Understanding how and why background uncertainty alters decisions can help to gain insight into how humans represent uncertainty and incorporate it into their decision making. For example, if risk preferences are constructed on the fly as suggested by some authors^1,2^, an enhanced comprehension of the impact of background uncertainty may help researchers to better understand this process. But before embarking on the endeavor of understanding how background uncertainty affects decisions at hand, it is paramount to ensure that the phenomenon actually exists, since previous findings are inconclusive.

This paper contributes to a deeper understanding of the phenomenon of background uncertainty through two key steps. Firstly, our literature review led us to the conclusion that background ambiguity has a more pronounced impact on risk aversion concerning the foreground decision than background risk. Secondly, we conducted four experiments to assess the robustness of the effect of background uncertainty on risk-taking behavior. Furthermore, we explored whether the type of background uncertainty (ambiguity vs. risk) determines the magnitude of the effect of background uncertainty. Overall, we did not find a meaningful effect of background uncertainty on risk-taking behavior. Only in one of our four experiments, we identified a small effect of background ambiguity on risk-taking behavior.

### Literature review: background uncertainty in economics and psychology

The literature in psychology and economics on the effects of background uncertainty is very heterogeneous in terms of its methods, particularly the type of background uncertainty investigated. At this point it seems necessary to distinguish between two types of background uncertainty which is often neglected in the literature. When the decision maker does not know the probabilities associated with the outcomes of the background event, we propose the term *background ambiguity*. Conversely, when the probabilities of the outcomes of the background uncertainty are known, we suggest using the term *background risk*.

### Related psychological literature

The effect of background uncertainty on risk-taking has not been tested explicitly in the psychological literature. The concept, however, is related to theories of risk compensation^3^ and risk homeostasis^4^. Both frameworks employ a threshold model and predict that individuals aspire to a specific level of risk. These theories have been used to explain the changes in human risk behavior with and without protective measures. When protective measures are used, perceived risk decreases leading to a more risk-seeking behavior, while the absence of protection increases risk perception, resulting in decreased risk seeking^5–10^. Importantly, predictions based on these theories focused solely on the risk associated with the decision at hand, neglecting background uncertainty.

The empirical evidence supporting the homeostasis hypothesis could be translated into economic terms as a substitution effect^11^. According to Quiggin^11^, if a certain level of background risk is inherent in the situation, it (partly) displaces the risk associated with the decision at hand.

### Related economics literature

Gollier and Pratt^12^ argued that risk preferences are affected by independent risks, and humans display higher risk aversion when background risk is present. A first test of Gollier and Pratt’s theoretical prediction was done by Harrison et al.^13^ in a field experiment with the visitors of a coin collector fair. The introduction of uncertainty about a coin’s value increased risk aversion in a lottery when payoffs were expressed in collector coins. As a conceptual replication of the field study at the coin fair, Herberich and List^14^ asked participants to make decisions either in a standard multiple price list lottery decision-making task^15^ or in one of three modified multiple price list lotteries similar to Harrison et al.^13^. Risk aversion was lower in the modified background uncertainty lotteries than in the standard lottery, but the effect was not significant.

Results from other studies support the hypothesis that background uncertainty increases risk aversion. Individuals who recently experienced floods or earthquakes showed higher risk aversion than individuals in similar areas who had not been affected^16^. This result is consistent with the observation that individuals became more risk-averse after experiencing a volcanic eruption than they were before^17^. Using socioeconomic survey data from Germany, Bacon, Conte, and Moffatt^18^ showed that risk aversion covaried with financial insecurity between 2004 and 2012. Particularly in 2008, risk aversion increased, which was regarded as evidence of the effect of background uncertainty on risk-taking behavior. A similar effect was observed using macro-economic data in the United States, where life-time experiences of external shocks were linked to a reduction in the investment in risky assets^19^.

The empirical findings reported above seem to support Gollier and Pratt^12^. However, in all studies referenced above, the probabilities of the outcomes of the background event were unknown to the decision maker. Thus, what these studies actually investigated was the effect of a specific form of background uncertainty that should be referred to more precisely as *background ambiguity.*

In contrast, when experimenters introduce *background risk*, the findings are less consistent. In a laboratory experiment, Lusk and Coble^20^ compared a control group, an unfair background risk group, and a fair background risk group to explore the effects of background risk. All participants completed lottery decisions in a multiple price list design, and participants in the background risk group were informed that there would be another lottery afterwards. In the fair background risk group, the subsequent lottery had a mean of zero, and participants had a 50% chance of winning an additional $10 or losing $10. In the unfair background risk group, the subsequent lottery had a mean of -$5, and participants had a 50% chance of losing $10 or otherwise losing nothing from a previously received endowment. Interestingly, background risk did not induce a change in risk-taking behavior. However, due to the experiment’s design, the second lottery was present only in the background risk groups, leading to different cumulated final payoffs. For this reason, two parameters reflecting risk aversion were compared to test the effect of final wealth, i.e., income.

The findings of Lusk and Coble^20^ differed depending on how the incorporation of the endowment from the background risk was modeled. Neither constant relative risk aversion, without considering the additional wealth from the lottery, nor constant absolute risk aversion showed an effect of background risk.

When comparing individuals’ risk preferences in an investment game, Beaud and Willinger^21^ found evidence of a background risk effect. Here, background risk was introduced by splitting a participant’s initial endowment into two accounts. Participants played the investment game for two rounds, and half of the endowment could be lost in one of the two rounds. Participants were informed that there was an equal likelihood that they would either lose or keep half of their endowment for that specific round. The results indicated that participants act more risk averse when their endowment was subject to background risk.

He and Hong^22^ utilized a multiple price list decision-making task where participants faced 12 lotteries. Three groups were compared, with the variances of the outcomes of six lotteries differing across groups, i.e., high, medium, or low. Participants completed all multiple price lists, and one decision was randomly selected in order to rule out learning and portfolio effects. Participants who completed the high-risk lotteries first demonstrated increased risk-aversion in later medium-risk and low-risk lotteries. This effect could also be attributed to background risk, as the increased variance of the unresolved lotteries carried over to influence subsequent decisions.

In summary, field studies have consistently provided support for the effect of background uncertainty, while results from laboratory studies have been mixed. The observed heterogeneity may be attributed to methodological differences between lab and field studies, specifically the type of investigated background uncertainty.

Orthogonal to the problem of the type of uncertainty, not all studies involving background uncertainty introduced its conception as (a) exogenous, (b) present during the decision-making process, or (c) beyond the control of the decision-maker. He and Hong^22^ examined the carry-over effect of decisions involving high-variance outcomes to decisions regarding options yielding medium-variance payoffs. While they identified an effect, it remained unclear whether the effect was due to background risk, given the risk associated with the initial decision was not exogenous and its outcome depended on the decision-maker.

A similar problem is shared by the field study of Willinger et al.^17^, where risk preferences were measured before and after background uncertainty was resolved, whereas risk preferences were not measured during the presence of background uncertainty. Lusk and Cobble^20^, on the other hand, informed participants that an additional lottery would be played after the decisions were made. Yet, this future event might not have been salient during the decision-making process. Conforming to the definition of background risk—requiring its presence and unresolved status during the decision-making process—the experiment by Beaud and Willinger^21^ stands out as the only study that observed an effect of background risk.

The current state of the literature calls for an experimental design that allows (a) the measurement of risk-taking behavior while participants are actively exposed to background uncertainty and (b) while systematically varying the information provided to participants, in order to test whether background ambiguity and background risk have different effects. The four studies presented here aimed at answering this call. In Table 1 we provide an overview of the treatments and measures implemented in our four studies, as well as the respective sample sizes and basic design choices.

**Table 1:** Overview of studies: manipulations and risk preference measures

	Background uncertainty		Risk preference measure						Design
	Risk	Ambiguity	LDT	BART	BRET	CEM	MPL	SCL	
Study 1	X	X	X						B/L
N=85									
Study 2	X	X		X					B/L
N=101									
Study 3		X			X	X	X	X	B/O
N=185									
Study 4	X	X				X	X	X	W/O
N=492									

*Note.* LDT = binary lottery decision task, BART = Balloon analogue risk task, BRET = Bomb risk elicitation task, CEM = Certainty equivalent method, MPL = Multiple price list, SCL = Single choice list; B = between subject design, W = within subject design, O = online study, L = lab study

## Results

**Fig. 1:**
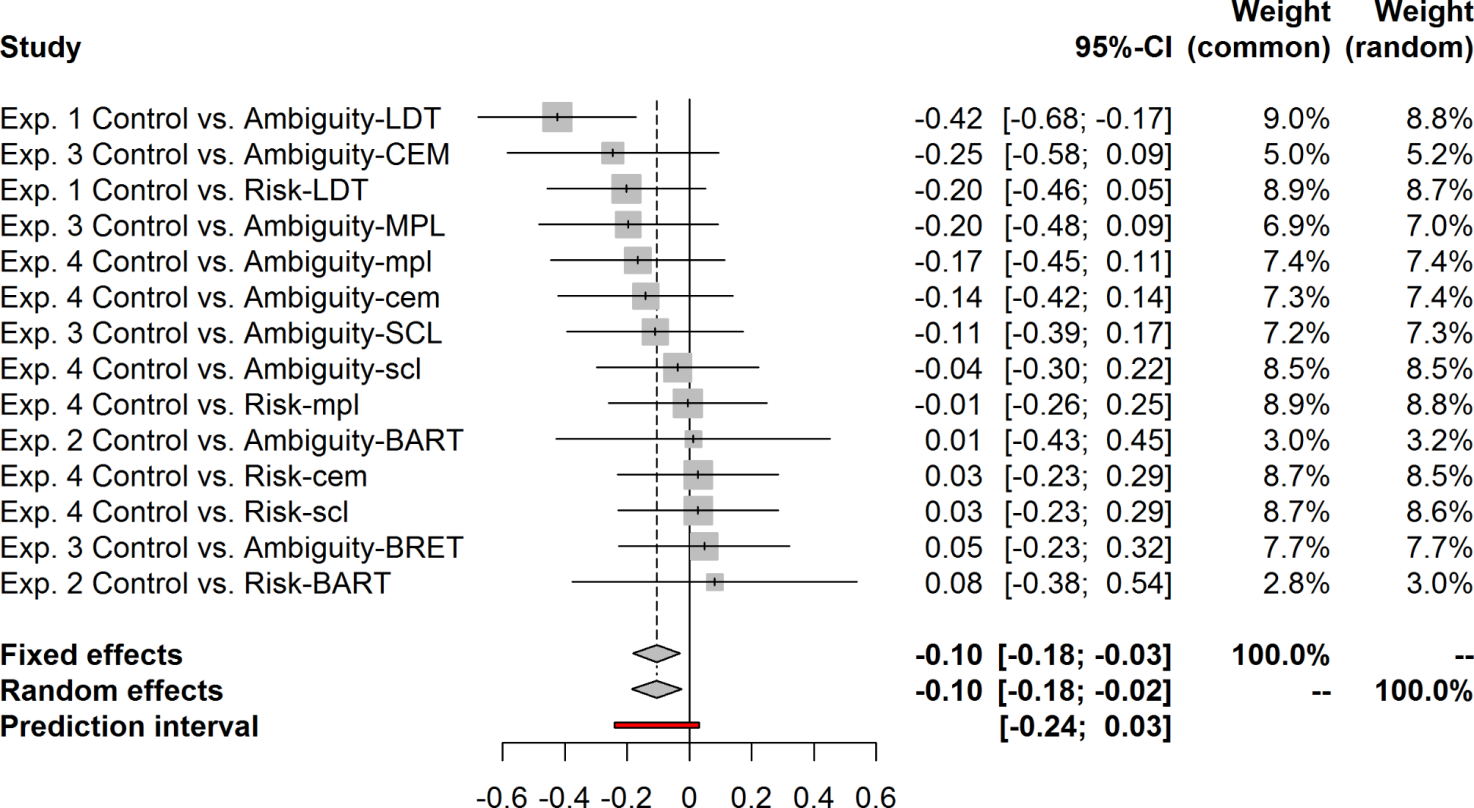
Forest Plot showing the results of the Fixed Effects and Random Effects for the Studies 1-4. Note. The analysis does not take into account the dependence of the control group in the multiple comparisons in Study 1 and Study 4. However, either dropping one comparison or removing the dependence by splitting the control groups was deemed not feasible as this would have resulted in small sample of the group and a change of the effects compared to the main analysis in the paper. For these reasons, the joint analysis should be treated as an exploratory tool to show the distribution of effects across all studies as the assumption OF the fixed and random effects models are violated.

In this section we start with a short overview of the main results followed by more detailed summary of the results for the particular studies. To estimate the effect of the two types of background uncertainty across all four studies, we carried out a joint analysis. We used the {esc} package^23^ to transform the effect sizes into Cohen’s *d* and the {meta} package^24^ for the meta-analysis. The results are shown in Figure 1. The random effects model showed an overall negative effect of background uncertainty on risk measures, *d* = -0.10, 95% CI [-0.18, -0.02], with background uncertainty resulting in higher risk aversion. At the same time Figure 1 also implies that there is no systematic difference between background risk and background ambiguity.

### Overview

In Study 1, participants had to make lottery decisions. We varied background uncertainty (control vs. risk vs. ambiguity) using a wheel of fortune that was spinning while participants made their decisions. The outcomes of the wheel of fortune determined a major part of participants’ payoffs. The results imply that people tend to be more risk averse in the presence of background risk and background ambiguity compared to situations where there is no background uncertainty. Risk taking behavior, however, does not differ between the two types of background uncertainty.

In Study 2, we used a wheel of fortune to induce background uncertainty (control vs. risk vs. ambiguity) and used the BART to measure risk preferences. We did not observe a significant effect of background risk or background ambiguity on risk-taking behavior.

In Study 3, we investigated whether the effect of background ambiguity depends on the measure that is used to elicit risk preferences. In an online study, we compared a group exposed to background ambiguity, employing four different measures of risk preferences, with a control group. Only in the MPL task, people choose less risky lotteries in the presence of background ambiguity.

In Study 4, our objective was to examine potential differences between background risk and background ambiguity concerning their effects on risk aversion. Additionally, we sought to determine whether the observed effects were similar or distinct across various measures. The results provide no evidence for an effect of background risk or background ambiguity on risk-taking behavior in any of the three measures (CEM, MPL and SCL).

### Study 1

In Study 1, we aimed to provide a first test of our conjecture that the two types of uncertainty - risk and ambiguity - would exert different effects on risk-taking behavior. To accomplish this, we experimentally manipulated the type of background uncertainty. We also tested for the moderating effects of framing the decision and the probability of winning.

Table 2 presents the regression results where the standard errors are clustered for each participant, and all interactions were included. The participants in the control treatment were less risk averse than the subjects in the background risk treatment, OR = 0.57, 95% CI [0.31, 1.05], and the background ambiguity treatment, OR = 0.38, 95% CI [0.21, 0.70]. The difference between the ambiguity and risk treatments was nonsignificant, OR = 0.63, 95% CI [0.39, 1.03].

**Table 2:** Effects of treatment, framing, and odds of winning on risky choices

Predictors	Odds ratios	95 % CI	*p*
Int.	1.20	[0.77, 1.89]	.419
Risk	0.57	[0.31, 1.05]	.073
Ambiguity	0.38	[0.21, 0.70]	.002
Framing	1.93	[1.47, 2.54]	<.001
Log-odds of winning	0.89	[0.80, 0.98]	.018
Side	0.95	[0.81, 1.11]	.491
Risk X framing	1.02	[0.70, 1.51]	.907
Ambiguity X Framing	0.90	[0.61, 1.32]	.598
Risk X log-odds of winning	0.94	[0.82, 1.09]	.418
Ambiguity X log-odds of winning	0.91	[0.79, 1.05]	.189
Framing X log-odds of winning	0.99	[0.85, 1.14]	.877
Risk X framing X log-odds of winning	0.98	[0.79, 1.20]	.819
Ambiguity X framing X log-odds of winning	1.07	[0.87, 1.31]	.508
	Random effects		
σ2		3.29	
τ00 VP_Code		1.16	
ICC		0.26	
N VP_Code		85	
Observations		3,240	
Marginal R^2^/Conditional R^2^		.080/.319	

*Note.* σ^2^ shows the within-subjects standard deviation. τ_00_ shows the between-subjects standard deviation. ICC indicates the intraclass correlation, that is, the proportion of variance between individuals (τ_00_) explained by the overall variance (σ^2^ + τ_00_). The marginal *R*^*2*^ provides the variance explained only by the fixed effects, and the conditional *R*^*2*^ provides the variance explained by the entire model (i.e., both fixed effects and random effects)

Participants were more risk averse in the gain than in the loss framing, OR = 1.93, 95% CI [1.47, 2.54], the proportion of choices in favor of the gamble was affected by the probability of the payoffs, and higher odds in favor of winning resulted in higher risk aversion, OR = 0.89, 95% CI [0.80, 0.98].

The results show, on average, risk aversion is higher when background uncertainty is present. Additionally, the type of background uncertainty influences the effect size. Background ambiguity increased risk aversion compared with the control treatment, and albeit background ambiguity did not differ significantly from background risk, background risk was not significantly different from the control treatment. Furthermore, background uncertainty did not alter the effect of framing or probability weighting. Our observation that the increase in risk aversion was affected by the type of uncertainty is in line with the finding that observed effect sizes in field studies investigating background ambiguity e.g.,^13^ were larger than the effects in the laboratory studies investigating background risk^14,20^.

One limitation of Study 1 is that the induction of background uncertainty was very similar to the decision task. To address this, we sought to conceptually replicate our study using a different task for a more comprehensive understanding.

### Study 2

Study 2 addressed the question of whether the effect of background uncertainty is task-independent in terms of the behavioral measure and the method that was used to induce background uncertainty. The preregistration of Study 2 can be found here: https://osf.io/v4ak8

In Study 2 we did not observe an increase in risk aversion in participants exposed to background uncertainty. One possible explanation is that the method used to induce background uncertainty does not influence tasks that are governed by more automatic processes, which are supposed to underlie decision making in the BART^25^. Alternatively, the sequential nature of the task and therefore the trial-by-trial learning^26,27^ might be stronger than the effect from background uncertainty. It has been observed that in lottery decisions where participants face a riskless alternative in the task, they behave more risk averse– a phenomenon known as the certainty effect of lottery decision^28^. It might be, that inducing background uncertainty rendered the riskless option even more attractive in experiment 1. Finally, it is possible that the sample size was too small resulting in low power to detect the effect of background uncertainty. To address the last two points, increasing power and addressing the possible effect of the task on the effect of background uncertainty, we conducted Study 3.

### Study 3

We carried out an online experiment in which participants responded to four measures of risk preferences, of which three used lottery decisions and one was based a direct behavioral measure. The preregistration of Study 3 can be found here: https://osf.io/gn395. Participants were assigned to one of two experimental treatments: a *background ambiguity* treatment or a *control* treatment. The latter received a fixed show-up payment of 4€. Participants in the ambiguity treatment were informed that, in addition to their base winnings, they would also receive 0€ or 8€ depending on a lottery that is played during the experiment. All participants completed all four measures of risk preferences. The treatment (control and background ambiguity) served as our first independent variable, whereas the measure of risk preferences was our second independent variable. If the effect of background ambiguity is similar across measures, there should be only a main effect of treatment. Otherwise, there should be an interaction between the measure and the presence of background ambiguity.

**Fig. 2:**
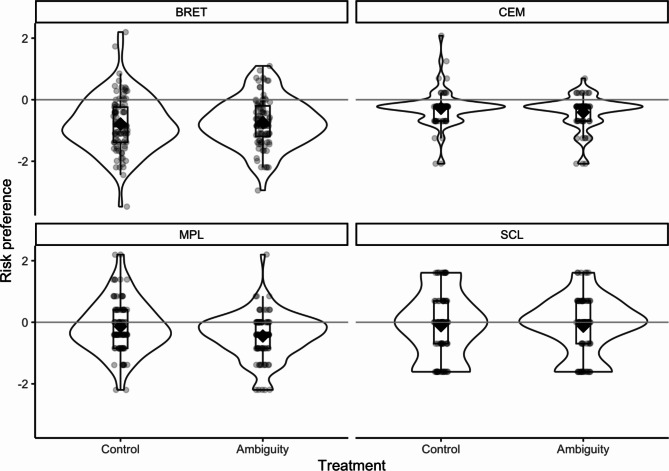
Comparing risk preferences across measurements and treatments. Note. The risk preference reflects the log ratio of choices in favor of the option with more risk. The horizontal line at y = 0 shows the value of risk preference given risk neutrality, that is, the ratio of risk/safe is 1 and the ln(1) = 0. The Violin plot depicts the density of the distribution. The boxplot shows the first, second (median), and third percentiles. The rhombus in each plot depicts the mean. The points are jittered individual responses.

As can be seen in Figure 2, participants were significantly more risk-averse in the BRET than in all other measures. To test the effect of the treatment on each measure, we ran a mixed-effects linear regression with the log ratio of risky choices as the dependent variable and a random intercept for each participant that accounted for the repeated measures. The treatment fixed effects and measures were included as dummy variables. The average risk aversion was higher in BRET than in MPL in the control group,* b* = -0.66, 95% [-0.88, -0.44], *p* < .001. The SCL,* b* = 0.04, 95% [-0.19, 0.27], *p* = .744, and CEM,* b* = -0.17, 95% [-0.42, 0.08], *p* = .178, were not significantly different from the risk aversion observed in the MPL.

There is a difference in risk aversion between the MPL control and the MPL ambiguity treatment, but there are no differences between the treatments for the other three measures. (for all coefficients, see Table 3).

**Table 3:** Regression results for risk preference regressed on treatment and measurement

Predictors	Estimates	CI	p
(Intercept)	-0.13	-0.31 – 0.04	0.144
Treatment [ambiguity]	-0.32	-0.57 – -0.07	0.012
task [bret]	-0.66	-0.88 – -0.44	<0.001
task [scl]	0.04	-0.19 – 0.27	0.744
task [cem]	-0.17	-0.42 – 0.08	0.178
Treatment [ambiguity] * task [bret]	0.36	0.05 – 0.68	0.022
Treatment [ambiguity] * task [scl]	0.29	-0.04 – 0.61	0.082
Treatment [ambiguity] * task [cem]	0.22	-0.13 – 0.57	0.220
Random effects			
σ^2^	0.56		
τ_00_ _participant_	0.13		
ICC	0.18		
N _participant_	185		
Observations	647		
Marginal R^2^ / Conditional R^2^	0.093 / 0.259		

*Note.* The regression was fitted using linear regression. The intercept reflects the response in the BRET and the control group. σ^2^ shows the within-subjects standard deviation. τ_00_ shows the between-subjects standard deviation. ICC indicates the intraclass correlation, that is, the proportion of variance between individuals (τ_00_) explained by the overall variance (σ^2^ + τ_00_). The marginal *R*^*2*^ provides the variance explained only by the fixed effects, and the conditional *R*^*2*^ provides the variance explained by the entire model (i.e., both fixed effects and random effects).

### Study 4

In Study 4, we implemented a within-subjects design to gain a more nuanced understanding of individual behavior. However, we deviated from a full-scale within-subjects approach where participants in a given experimental treatment would typically pass through all three experimental conditions—background ambiguity, background risk, and a control condition without any background uncertainty. To strike a balance between obtaining comprehensive data and minimizing the cognitive burden for participants, we conducted experimental treatments consisting two sequential *parts*. In the two parts of a given experimental treatment, participants were exposed to only two out of the three conditions. This decision was guided by the intent to reduce cognitive load and participant fatigue, which are critical factors in the reliability of behavioral measures. Furthermore, we chose to measure risk aversion using only three different measures, deliberately omitting the bomb risk elicitation task (BRET) to reduce the time participants had to spend on the study.

This adapted approach allows us to measure within-participant variation and examine how it is affected by the degree of risk aversion. By assessing risk aversion with multiple measures and across varying conditions, we can distinguish between random within-participant variation and systematic variation due to changes in background risk.

**Fig. 3:**
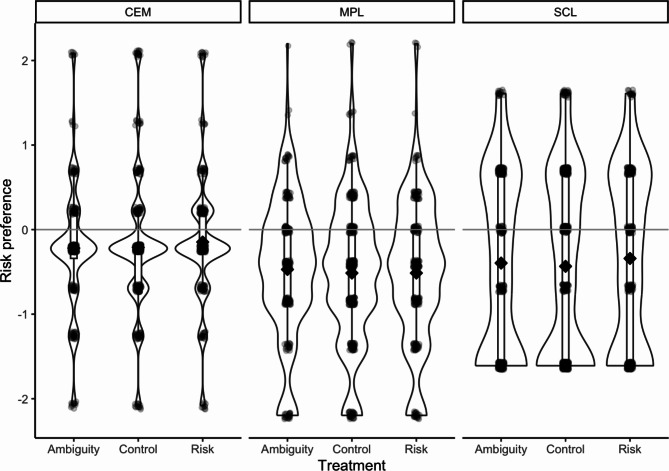
Comparing risk preferences across conditions and measurements

As can be seen in Figure 3 and Table 4, participants were significantly more risk-averse in the Certainty Equivalent Method (CEM) than in the two other measures. To test the effect of the experimental conditions on each measure, we ran a mixed-effects linear regression with the log ratio of risky choices as the dependent variable and a random intercept for each participant that accounted for the repeated measures. The fixed effects of the conditions and measures were added as dummy variables.

**Table 4:** Regression results for risk preference regressed on condition and measurement

Predictors	Model 1			Model 2		
	DV: log_ratio_risk			DV: log_ratio_risk		
	Estimates	CI	p	Estimates	CI	*p*
(Intercept)	-0.24	-0.37 – -0.11	**<0.001**	-0.26	-0.41 – -0.12	**<0.001**
condition [control]	-0.01	-0.10 – 0.08	0.789	0.01	-0.12 – 0.15	0.865
condition [risk]	0.05	-0.04 – 0.14	0.322	0.07	-0.07 – 0.22	0.316
task [mpl]	-0.31	-0.38 – -0.23	**<0.001**	-0.26	-0.40 – -0.11	**0.001**
task [scl]	-0.16	-0.23 – -0.08	**<0.001**	-0.14	-0.28 – -0.00	**0.049**
round number	0.01	-0.05 – 0.08	0.662	0.02	-0.05 – 0.08	0.632
condition [control] × task [mpl]				-0.05	-0.23 – 0.14	0.634
condition [risk] × task [mpl]				-0.10	-0.30 – 0.11	0.360
condition [control] × task [scl]				-0.03	-0.21 – 0.15	0.738
condition [risk] × task [scl]				0.00	-0.20 – 0.20	0.979
Random effects						
σ^2^	0.62			0.62		
τ_00_	0.22 participant			0.19 participant		
τ_11_				0.00 participant.conditioncontrol		
				0.00 participant.conditionrisk		
ρ_01_				1.00		
				1.00		
ICC	0.26			0.26		
N	489 participants			489 participants		
Observations	2435			2435		
Marginal R^2^ /Conditional R^2^	0.019 / 0.272			%1.%2 0.272		

While there is a difference between tasks across conditions, there is no difference between the conditions across tasks. Model 1 in Table 4 shows that the average risk aversion across conditions was higher in CEM than in MPL,* b* = -0.31, 95% [-0.38, -0.23], *p* < .001 and in SCL *b* = -0.16, 95% [-0.23, -0.08], *p* < .001. Contrary to that, there is no significant effect from the risk or the ambiguity condition. In Table 4 we use the ambiguity condition as the baseline to include both tests - control vs. ambiguity and risk vs. ambiguity – into the model. The control condition,* b* = -0.01, 95% [-0.10, 0.08], *p* = .789, and risk condition, *b* = 0.05, 95% [-0.04, 0.14], *p* = .322, were not significantly different from the risk aversion observed in the ambiguity condition.

These results are confirmed in Model 2 in Table 4 where we included interaction terms for the four combinations of the treatment conditions (control and risk) and measurement tasks (MPL and SCL). None of the four interactions shows a significant difference compared to the baseline CEM in the ambiguity condition.

## Discussion

We reviewed psychological and economics research that investigated the effect of background uncertainty on risk preferences. While some studies found an effect, others did not. We argue that two types of background uncertainty should be distinguished: background risk and background ambiguity.

We conducted four studies and tested how consistently background uncertainty altered risk preferences and whether the type of background uncertainty mattered. In two of four studies, we find only weak support for our hypotheses. We found some effects of background uncertainty in Study 1 and to some extent in some conditions of Study 3, while the other two studies do not show any effect.

Our findings suggest that risk preferences, revealed through binary choices, are not solely a function of those options, but they are also affected by the context and are not just properties of the options themselves, like framing effects are^29^.

In psychology, it is assumed that traits are stable, but behavior in which they are manifest has a certain degree of variation, which is caused by variations in the situation^30,31^. Our findings support this general assumption of variation in behavior given stable traits in psychology but oppose the frequently held assumption in economics that risk preferences are stable over time which should result in stable choices. Thus, our findings are in line with the recent proposition by economists that risk preferences should be considered as parameters in a distribution affected by situational factors^32^.

It has been assumed that the decision processes that underlie people’s choices might depend on the task used^33,34^. This fact may explain why some measures are more likely than others to be affected by background uncertainty. While lottery decisions may be quite sensitive to small changes in underlying risk preferences, behavior in the real world might be less so. The effects from Study 1 can be explained by the fact that the expected values of the binary options were equivalent. As a result, a small change in the risk preference led to qualitatively different choices. We also see this effect in the multiple price list decision in Study 3, where background uncertainty shifted the choices over one row, which is a small but consistent effect across participants. In both tasks, the decision involved clearly defined probabilities and consequences, which might be sensitive to the context. The decisions in the BART or BRET in Studies 2 and 3, respectively, were not influenced by the context, which might have been the case because the options were less clearly defined, and the decision-making task was repeated. This may in turn mean that participants might become more immersed in the task, or the task provided a more fine-grained measure of risk preference.

Furthermore, our findings in Study 3 show, consistent with Holzmeister and Stefan^35^, that risk preferences differ between measures. Our results also indicate that the effect of background uncertainty differs between measures, i.e., different measures yield different background uncertainty elasticities. Future research should investigate whether decisions between a lottery and a safe payoff with the same expected value are particularly susceptible to effects of background uncertainty.

It has been shown that uncertainty alters choice by altering the underlying neurological processes^36^. So far research has mostly focused on the uncertainty inherent in decision options and has found that it increased risk aversion^37^. We add to this research by showing that uncertainty in the environment also increases risk aversion. The influence of such background uncertainty may be explained in two ways: It can alter the reward system, which could be an effect at the neuronal level^38,39^, or it can influence risk preferences on a more general level by altering the information processed in the endocrine system. In this vein, it has been observed that individuals exposed to high levels of environmental uncertainty have high levels of cortisol, which should in turn result in higher risk aversion^40^. Future research could use the concept of background uncertainty to move toward a better understanding of how and when choices are altered by specific or more general neurological processes.

### Limitations

Our interpretation of the results rests on the assumption that our manipulation actually induced a heightened feeling of uncertainty. However, we did not measure for whether this was the case.

We did not examine the effect of unfair background uncertainty or uncertainty that could result in real losses. Background uncertainty in field studies was characterized by the possibility of losing due to natural disasters, and unfair background risk in the laboratory was induced through skewed payoff distributions with a higher likelihood of losses^20^. Thus, in such previous studies, a significant effect of background risk was found. It is possible that our manipulations in Studies 3 and 4 were too weak to elicit a response as strong as in Study 1.

We should also note that the different measures of risk preference used in the four studies presented have varying degrees of ambiguity due to their design. In the Certainty equivalent method (CEM), multiple price list (MPL) and single choice lottery (SCL), participants have complete information about the probabilities. In two other tasks (Balloon Analogue Risk Task, BART and the bomb risk elicitation task, BRET), the probabilities were not directly communicated to the participants. While the participants in the BART were told that balloons will explode after 128 pumps at the latest, the participants in the BRET knew that there are at most 99 safe boxes (with a total of 100 boxes and 1 bomb) but the actual probabilities were not communicated. In both measures, the participants therefore decided under ambiguity rather than risk. Variation in primary ambiguity of the tasks themselves could interact with background ambiguity, although the data from our current studies do not show this.

Future research should strive to measure a wider range of background uncertainty variations and use strong induction methods, such as background lotteries, which involve losses.

### Conclusion

We reviewed research from economics and psychology on effects of background uncertainty on risk-taking behavior. We argue that heterogeneity in observed effects in the literature is due to the different types of background uncertainty investigated in the studies, namely background risk and background ambiguity. In this paper, with four studies, we tested whether background uncertainty breeds risk aversion and whether the type of uncertainty moderates that effect. Our results suggest a small but consistent positive effect of background uncertainty on risk aversion, and the type of background uncertainty seems to moderate the observed effects: Background ambiguity as compared with background risk induced greater increase in risk aversion. We also found that measures of risk preferences differed regarding their background uncertainty elasticity as some measures showed strong change when uncertainty was added, whereas others were more robust.

## Methods

### Study 1

#### Participants and design

Students (*N* = 85, 71.8% women, M_age_ = 23.42 years with *SD*_age_ = 3.45) were recruited at the local university campus and participated in a decision-making experiment. All participants made repeated decisions between a lottery and a certain payoff in a computerized lottery decision-making task.

The study had a 3 x 2 x 9 mixed design. Participants were randomly assigned either to the control treatment (*n* = 28), the background risk treatment (*n* = 29) or the background ambiguity treatment (*n* = 28). All participants completed the binary decision task. Across trials, the framing (gain vs. loss) and probability for the high lottery payoff (winning) was varied (nine different probabilities). The number of choices in favor of the lottery was used to measure risk aversion.

#### Experimental treatments

In both background uncertainty treatments, participants received an endowment of 10€ and were informed, that while they completed the decision-making task, a lottery based on a wheel of fortune would run to determine whether they will keep or lose the 10€ at the end of the experiment. To control for wealth effects in the control treatment, participants were endowed with 5€ and were told that they would receive this payoff additionally to the payoffs that are contingent on their decisions. Hence, the expected value of the additional endowment was 5€ in all treatments.

In both treatments, the wheel of fortune spun while participants made their decisions in the lottery task. Participants were informed at the beginning of the experiment, that if the wheel stopped on green, they would keep the 10€ as additional payoff and if it stopped on red, they lose the 10€.

The difference between both uncertainty treatments was the information about the distribution of red and green areas on the wheel of fortune. Participants were shown the wheels of fortune before the start of the lottery task. In the background *risk* treatment, participants could see that 50% of the area was green; thus, the probability of winning was 50%. In the background *ambiguity* treatment, subjects could not see the actual size of the green or red area on the wheel of fortune because the wheel was covered with a screen that only allowed to see a small area through a cutout. Hence, these participants did not know the probability of winning.

The framing of the decisions varied within participants, i.e., loss or gain. In the gain frame, participants were informed that their initial endowment was 0€. They faced the decision between a guaranteed payoff of 5€ and a lottery with a positive expected payoff. In the loss framing, participants received an endowment for each trial. They faced a decision between a sure loss of *x* ($endowment-x =5\text{€}$) and a lottery with an expected loss.

The probability of winning varied within participants. The probabilities of the two outcomes of each lottery added up to one. To cover the low, medium, and high ranges, the probability that the lottery pays the winning payoff had nine different values. Table 5 shows all trial information that was presented to the participants.

**Table 5:** Information presented to the participants for each trial depicting endowments and payoffs for the sure option and each lottery for the gain and loss framings

Framing	Endowment	Sure option	Lottery			
			Outcome 1 (winning)			Outcome 2 (losing)
			Payoff	With p	Payoff	With 1- p
Loss	100	-95	0	0.05	-100	0.95
	50	-45	0	0.1	-50	0.9
	25	-20	0	0.2	-25	0.8
	15	-10	0	0.333	-15	0.667
	10	-5	0	0.5	-10	0.5
	7.5	-2.5	0	0.667	-7.5	0.333
	6.25	-1.25	0	0.8	-6.25	0.2
	5.55	-0.55	0	0.9	-5.55	0.1
	5.25	-0.25	0	0.95	-5.25	0.05
Gain	0	5	100.0	0.05	0	0.95
	0	5	50.0	0.1	0	0.9
	0	5	25.0	0.2	0	0.8
	0	5	15.0	0.333	0	0.667
	0	5	10.0	0.5	0	0.5
	0	5	7.5	0.667	0	0.333
	0	5	6.3	0.8	0	0.2
	0	5	5.5	0.9	0	0.1
	0	5	5.3	0.95	0	0.05

#### Measure

To measure participants’ degree of risk aversion, we utilized the *lottery decision task* employed by de Martino, Kumaran, Seymour, and Dolan^29^. In each trial, participants were informed about their endowment for this trial and faced a binary choice where they had to decide between a safe option and a risky option (a lottery with two probabilistic outcomes). Half of the trials were in the gain frame and the other half were in the loss frame. The probabilities were depicted numerically and as pie charts. Participants completed all decisions twice, to control for the left and right positioning of the lottery on the screen, thus participants completed 36 choices.

The dependent variable (risk aversion) was measured as the number of choices in which the participants preferred the lottery over the sure payoff. At the end of the experiment, one of the decisions was randomly selected and played out.

#### Procedures

Participants were recruited via social media posts in various university groups, billboards, direct instant messaging, email, or direct face-to-face conversations. After signing up online for a testing session, all participants completed an online version of the Emotion Regulation Questionnaire^41^ at least 24 hours. before the laboratory session took place. In the laboratory, participants were tested in groups of one or two. They were seated in individual cubicles and received written instructions. In each session, we tested participants from the same experimental treatment. The instructions were standardized and differed only in the treatment-specific parts. When participants had questions about the procedure, they were allowed to ask the experimenter. Participants were informed that their decisions would determine one part of their compensation. Furthermore, participants in the control treatment were informed that they would receive an additional payoff of 5€, which was placed in front of them. In the background uncertainty treatments, 10€ was placed in front of the participants, and they were told that they could either keep or lose the 10€ depending on whether the marker on the wheel of fortune landed on a green or red area. When participants were ready, the wheel of fortune was spun, and participants completed the computerized decision task. When participants signaled that they had finished the decision task, the wheel of fortune was stopped by the experimenter, and the position was recorded. Then one decision made in the decision-making task was randomly selected and played out. Participants were then paid and left the laboratory.

### Study 2

#### Participants and design

The number of participants was determined on the basis of the assumptions that each participant would perform the BART 30 times according to the standard procedure and that the retest reliability of the BART is .77^42^. We aimed to achieve a power of .9 and expected an effect size of f = .3 on the basis of our Study 1. The sample size calculated for an ANOVA with repeated measures and a between-subjects factor with three types calculated with G*Power^43^ was N = 102. Participants were recruited via ORSEE from the local participant pool (Greiner, 2015). Because one participant had to be discarded due to a computer crash, we had a final sample of N = 101 (43.6% women, M_age_ = 24.29 years, SD_age_ = 6.56). Participants were randomly assigned to one of three treatments; control (n = 33), background risk (n = 34), or background uncertainty (n = 34).

#### Experimental treatments

We used the same experimental manipulation of background risk and background ambiguity as in Study 1 by using a wheel of fortune, partitioned into green and red sections. Participants in the ambiguity treatment did not see the full wheel and did not know how the red and green areas were distributed. Participants in the risk treatment saw the distributions. The winning price of the lottery was an additional amount of 10€. Participants had already received the 10€ at the beginning of the experiment and either lost it, if the wheel landed on read or kept it if the wheel landed on green. Participants in the control treatment received a fixed amount of 5€, and the wheel had no consequences for them.

#### Measures

Risk preferences were measured using the decisions in the Balloon Analogue Risk Task (BART). Participants had to inflate a virtual balloon with repeated “pumps”^42,44^. Participants knew that the balloons could burst after a certain number of pumps but not at what point the ballon will burst. Unknown to participants, the maximum number of pumps was 128. In line with previous research, the maximum number of pumps per balloon was randomized once before the experiment and remained the same for all participants to avoid variability across groups^27,42,44,45^. Participants received 0.05€ per pump. After each pump, they could decide whether to stop pumping and cash in their accumulated payoff or to continue pumping. If the balloon bursts, all payoffs that had accumulated for that balloon were lost.

#### Procedures

After signing up online for a testing session, all participants completed an online version of the ERQ^41^ and the NEO FFI^46^ at least 24 hours before the laboratory session took place. In the laboratory, participants were tested in groups of 8 to 12. They were seated in individual cubicles and received written instructions. All participants in a particular session were assigned to the same experimental treatment. The instructions were standardized and differed only in the treatment-specific parts. If participants had questions about the procedure, they were allowed to ask the experimenter. Participants were informed that their decisions would determine one part of their compensation. Furthermore, participants in the control treatment were informed that they would receive an additional payoff of 5€, which was placed in front of them.

In the background uncertainty treatments, 10€ were placed in front of the participants, and they were told that they could either keep the 10€ or lose the money depending on whether the wheel of fortune landed on a green or red area. When participants were ready, the wheel of fortune was spun, and participants completed the computerized decision task. When participants signaled that they had finished the task, the experimenter stopped the wheel of fortune, and the position was recorded. In the end, participants saw their final number of pumps, the resulting payoff, and the additional payoff they received from the background lottery. They were then paid and left the laboratory.

### Study 3

#### Participants and design

We planned to recruit 90 participants per treatment for a total N of 180. The power calculation was based on the simplistic assumption of a repeated-measures design with a power of .8 with an alpha of .01 for an effect of *f* = 0.2 in a repeated-measures ANOVA design (2 x 4) given a correlation of .5 between measures (GPower)^43^.

Participants were recruited via ORSEE^47^ from the local participant pool. Students *N* = 185 (70.8% women, *M*_age_ = 24.25 years, *SD*_age_ = 5.41) were randomly assigned to one of the two experimental treatments (control *n* = 93, background ambiguity *n* = 92). The experiment was conducted online using oTree^48^.

#### Experimental treatments

In the background ambiguity treatment, participants were told that at the end of the experiment, they would receive either an additional 8€ or nothing depending on a random mechanism that was visualized by the shifting logos of the universities at the top of the page. If the logo of the participant’s university was on the right-hand side when they got to the final payoff page, the participant would receive the payoff; if the logo was on the left-hand side, their additional payoff would be zero. The likelihood that the logos would switch was unknown to the participant, and the logos switched at irregular intervals. In the control treatment, the logos switched sides in the header too, but subjects were not informed about the relevance of these switches.

#### Measures

Risk preferences were measured with four different methods utilizing the ready-made applications for Otree by^49^. We used the measures and followed the same procedures as^35^, but downscaled the payoffs by a factor of 0.5. We utilized the bomb risk elicitation task (BRET), certainty equivalent method (CEM), multiple price list (MPL), and single choice lottery (SCL). In the following,${\varvec{x}},{\varvec{p}};{\varvec{y}}$ denotes a two-outcome lottery that assigns probability ${\varvec{p}}$ to outcome ${\varvec{x}}$ and probability $1-{\varvec{p}}$ to outcome ${\varvec{y}}$. Subscripts ${\varvec{h}}$ and ${\varvec{l}}$ refer to “high” and “low” lottery outcomes.

The BRET is a visual risk preference elicitation method requiring participants to decide how many boxes to collect out of a matrix containing $n$ boxes. Each box they collect yields a payoff γ. In one of the boxes, however, there is a “bomb” erasing all previous earnings. Thus, potential earnings increase linearly but are zero if there is a bomb in one of the collected boxes. The BRET elicits (within-method) consistent decisions in $n + 1$ lotteries $\left(\gamma k,\frac{n-k}{n}; 0\right).$ The number of boxes collected is a single parameter $k \in \{0, 1, . . ., n\}$, which reflects a participant’s risk aversion. We used the dynamic version of the BRET (Crosetto & Filippin, 2013), where after pressing the start button, boxes are collected automatically until the stop button is pressed. The location of the bomb was revealed only at the end of the task. In our experiment, we set $n = 100$ (i.e., 100 boxes are displayed) and $\gamma = 0.25$€ (i.e., participants gain 0.25€ per box), implying an expected payoff of 6.25€ for a risk-neutral decision-maker.

The CEM determines the point of indifference between a lottery ${L}_{a} = ({h}_{a}, p; {l}_{a})$ with ${h}_{a}> {l}_{a}$ and $n$ varying degenerated lotteries (lotteries with one sure outcome) where the payoff changes in each step $i$, ${L}_{b,i} = ({b}_{i}, 1)$, with ${h}_{a} \le {b}_{i} \le {l}_{a}$ for all $i = 1, 2, . . ., n$. We implemented the parametrization used by Abdellaoui et al. (2011), with $n=9$ binary choices, scaled by a factor of .25; hence, ${h}_{a}= 7.50\text{€}, {l}_{a}= 2.50\text{€}$, and ${b}_{i}= \left\{2.50\text{€}, 3.125\text{€}, . . ., 7.50\text{€}\right\}$. A risk-neutral participant can expect to earn 5.695€.

The MPL is characterized by a menu of 10 binary choices. In each choice, two lotteries are depicted with fixed payoffs but varying probabilities of high and low outcomes for each option. Hence, participants face multiple binary choices $i$ between lottery ${L}_{a} = ({h}_{a}, {p}_{i}; {l}_{a})$ and lottery ${L}_{b} = \left({h}_{b}, {p}_{i}; {l}_{b}\right)$ for $i = 1, 2, . . ., n$, where ${h}_{b}> {h}_{a}> {l}_{a}> {l}_{b}$. We used the parametrization with *n* = 10 lotteries as proposed by^50^ but scaled the payoffs by a factor of 2.5, which resulted in ${h}_{a}=5.00\text{€}, {l}_{a}=4.00\text{€}$, and ${h}_{b}=9.625\text{€}, {l}_{b}=0.25\text{€}$ with ${p}_{i}= \{0.10, 0.20, . . ., 1.00\}$. A risk-neutral individual can expect to earn 6.07€. To avoid the problem of multiple switches, we enforced consistency and did not allow participants to switch back and forth.

The SCL offers participants a menu of different lotteries, asking them to choose the one they prefer to be played out. The menu consists of six lotteries that are similar to the implementation proposed by^51^ and simplified by^52^. The payoffs were: $$L1 = (4.50\text{€}, 0.50;$$$$4.50\text{€}), L2 = (3.75\text{€}, 0.50; 6.00\text{€}),$$$$L3 = \left(3.00\text{€}, 0.50; 7.50\text{€}\right), L4 = \left(2.25\text{€}, 0.50; 9.00\text{€}\right),$$$$L5 = (1.50\text{€}, 0.50; 12.00\text{€}),$$ and $$L6 = (0.00\text{€}, 0.50; 12.00\text{€})$$. Lotteries L5 and L6 had the same expected payoff but different standard deviations. That is, choosing L5 implies that the decision-maker is either (weakly) risk-averse or risk-neutral; choosing L6 reveals risk neutrality or risk-seeking preferences. Hence, a risk-neutral decision-maker opts for either lottery L5 or lottery L6, implying an expected payoff of €6.00. One of the tasks was randomly chosen and determined a part of the final payoff for each participant. The order in which the tasks were presented was randomized to avoid order and learning effects.

The tasks differed in the number of possible risky choices and the payoffs, but in all tasks, participants switched from risky options to safer options (or from safe to risky) at some point in the task. For this reason, the ratio of risky to safe choices in each measure was an empirical measure of the participant’s risk preference. Because ratio measures have a lower bound of zero and an upper bound of infinity, we log-transformed them so that they were linear and followed a normal distribution with a mean of zero in the case of risk neutrality (i.e., the same proportion of risky to safe choices). We did not preregister this transformation as we overlooked this problem of comparability when designing the study.

After making their decision in the respective task, participants were shown their decision and asked about the perceived risk of their decision and their confidence in their choice. We did not include these measures in the analysis as they were only for exploratory purposes.

#### Procedures

After signing up online for a testing session, all participants completed an online version of the ERQ^41^ and the NEO FFI^46^ at least 24 hours before the online experiment took place. These questionnaires were part of a separate research project. Participants received a second email with a personalized link to the online experiment, and had to enter a code they received after completing the ERQ and NEO FFI survey. Participants in the control treatment were informed that they receive a show-up payment of 4€. In the background ambiguity treatment, participants were told that, in addition to their earnings, they would receive either an additional 8€ or no additional payoff, depending on the position of their university’s logo at the top of the screen. Each task commenced with instructions followed by comprehension tests. At the end, one of the tasks was randomly selected, one randomly chosen decision was played out, and participants were paid accordingly. Here, participants saw their decision, the resulting payoff, and the additional payoff they received from the background lottery or the 4€ show-up payment. Participants were given Amazon gift cards with the corresponding values of their payoffs.

### Study 4

#### Participants and design

The preregistration for Study 4 is found here: https://aspredicted.org/DPF_LXZ. We carried out simulations to estimate the power for the between subject comparison of an interaction effect between part and treatment, which is the statistical test of a difference of change. We fit the following model:

$${y}_{i,part} = {\beta }_{\left\{0\right\}}+ {\beta }_{\left\{1\right\}}*Control + {\beta }_{\left\{2\right\}}*Ambiguity + {\beta }_{\left\{3\right\}part2}+ {\beta }_{\left\{4\right\}part2}*Control + {\beta }_{\left\{5\right\}part2}*Ambiguity$$

beta0 = -.12 Risk aversion before treatment in the risk group; beta1 = 0 Risk aversion before treatment in the control compared to risk group; beta2 = 0 Risk aversion before treatment in the ambiguity compared to risk group; beta3 = -.5 Risk aversion after treatment in the risk group; beta4 = 0.5 difference of Risk aversion after treatment in the control compared to risk group; beta5 = -0.3 difference of Risk aversion after treatment in the ambiguity compared to risk group; sigma0 = .15

We found that at least 90 % power for the between subject effect was obtained with 95% CI given a sample size of N = 204 (n = 68 per treatment). These results would also be sufficient to the results from our previous studies. As a safeguard against potentially incomplete observations (e.g., participants who drop out before completing the experiment), we aimed for a sample size of n=70 per treatment (N=490), the final sample was N = 492.

Participants were recruited through Prolific and experienced two of the three experimental conditions. The order of conditions was counterbalanced across participants using a series of treatment combinations, ensuring that each participant encountered a unique sequence of conditions. This counterbalancing was derived from a reduced Latin square design, adapted to accommodate the two-condition structure, resulting in seven distinct treatment orders (see Table 6).

**Table 6:** Reduced latin square and resulting treatment order

Treatment	Condition 1	Condition 2	N
T1-CC	Control	Control	71
T2-CR	Control	Risk	72
T3-CA	Control	Ambiguity	71
T4-RA	Risk	Ambiguity	70
T5-AR	Ambiguity	Risk	69
T6-RC	Risk	Control	69
T7-AC	Ambiguity	Control	70

The experiment was conducted online using the oTree platform^48^. Each participant completed all three behavioral tasks designed to measure risk preferences—Certainty Equivalent Method (CEM), Single Choice List (SCL), and Multiple Price List (MPL)—in each of the two conditions they were assigned to. The order of these three tasks was consistent across both conditions for each participant and was the same on both occasions, but the sequence in which participants encountered the conditions was randomized to control for any order effects.

#### Experimental treatments

As stated above, each experimental treatment consists of two conditions. In the *background ambiguity condition*, participants were endowed with a sum ($e$) and told that a concurrent lottery could result in an outcome of either $-e$ or $+e$ (resulting in an outcome of either $0$ or $2e$, effectively doubling their endowment or reducing it to nothing). This lottery was depicted as a wheel of fortune with green and red sections. To introduce ambiguity, the exact distribution between the green and red sections was not disclosed to the participants. Before the risk assessments, they were shown 11 different potential distributions of the wheel, ranging from fully red to fully green, each spinning and then halting to illustrate the lottery mechanism. During their decision-making, the wheel was concealed by a gray cover, showing movement but not the specific sections, to maintain the element of uncertainty. This operationalization of ambiguity has its limits, which should be acknowledged here. In contrast to the background risk condition (see below), participants in the ambiguity treatment were only aware that the probability was drawn from a uniform distribution. This maintains first-order ambiguity, as participants do not know which specific probability was chosen. However, it eliminates second-order ambiguity because participants know the set of possible probability distributions and the probability with which these distributions occur. This second-order certainty was a necessary trade-off. Not giving information about possible probabilities could reduce the perceived uncertainty: without information about the distribution, participants might assume that a 50:50 outcome is most likely.

For the *background risk condition*, participants received the same initial endowment and faced a similar lottery. However, they were informed that the wheel of fortune is equally divided in a green and a red section, symbolizing a balanced risk with a 50-50 probability. Unlike in the ambiguity condition, participants could see the colored sections as the wheel spun while they made their decisions.

In the *background neutral control condition*, the procedure was identical to the risk condition with the exception that participants were assured of a no-loss lottery, effectively creating a neutral background. They were informed that the outcome would be based on a fully green wheel, indicating that their endowment would be returned regardless of the wheel’s spin.

Consistency was maintained across all conditions with the spinning wheel being displayed next to the decision-making tasks. The wheels were always in motion during the decision phase to simulate the ongoing lottery, but they were not brought to a stop until the final page, where the payoffs based on the lottery outcomes were revealed.

#### Measures

Risk preferences were again measured with three different methods utilizing the ready-made applications for Otree by^49^. We utilized the certainty equivalent method (CEM), multiple price list (MPL), and single choice lottery (SCL). For a detailed description of the three measures see the Measures Section of Study 3.

#### Procedures

**Fig. 4:**
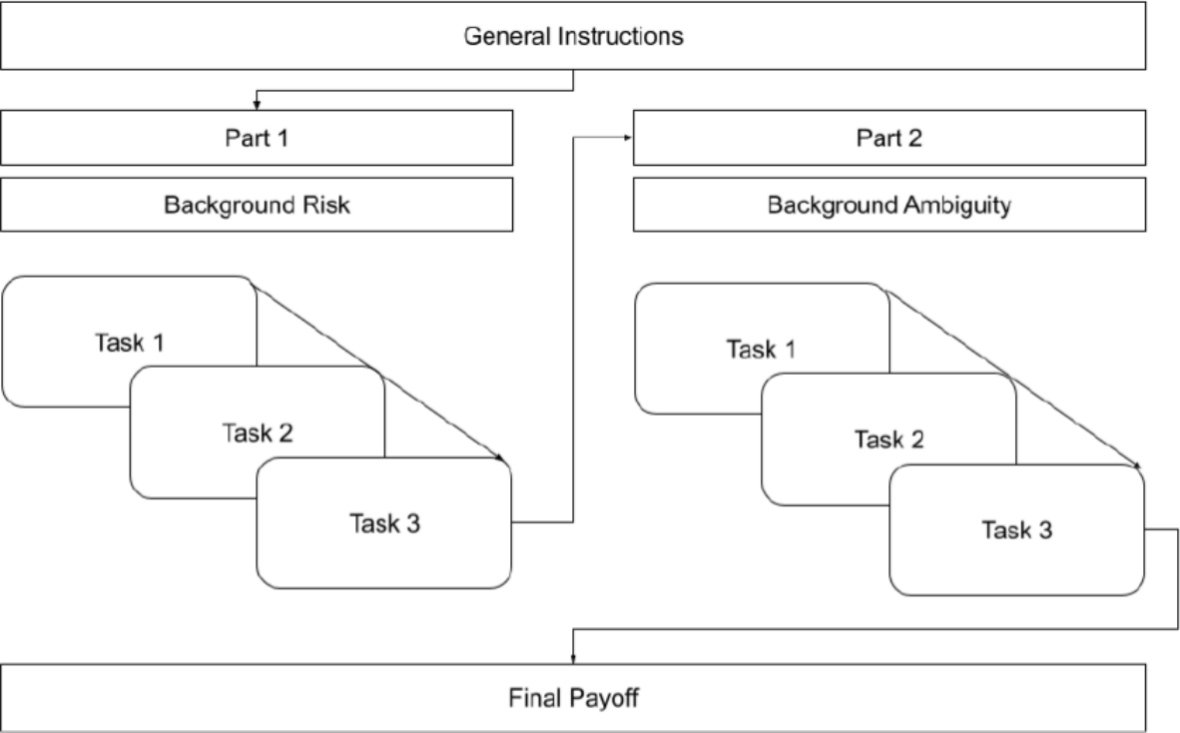
Experimental procedure showing sequence of conditions for the treatment T4-RA as an example (part 1: background risk and part 2: background ambiguity)

Participants were briefed that the experiment consisted of two distinct parts, each containing a set of three decision-making tasks. Each part corresponded to a different experimental condition, with Part 1 and Part 2 each representing one of the two conditions. Within each part, participants would engage in the three risk preference tasks described above, with the sequence of tasks being randomized at the beginning of the experiment for each participant. This sequence remained fixed for the duration of the experiment to ensure consistency. Figure 4 visualizes the sequence of events exemplary for the treatment T4-RA, with the first condition being background risk, and the second background ambiguity.

Participants received sequential instructions for each part accordingly. The experimental compensation was structured around a bonus payoff system that was dependent on the decisions made during these tasks. A random round payoff mechanism was used to determine earnings, similar to the approach by^21^. After completing both parts, one part was randomly selected to be payoff relevant. Within the selected part, one task was also chosen at random, and the participant’s bonus was calculated based on the outcome of this task, combined with the result of the background lottery associated with the payoff relevant part.

This approach ensured that each decision within each part was potentially consequential, thereby motivating participants to treat each choice with equal seriousness, as any decision could ultimately impact their final payoff^53^.

**Fig. 5:**
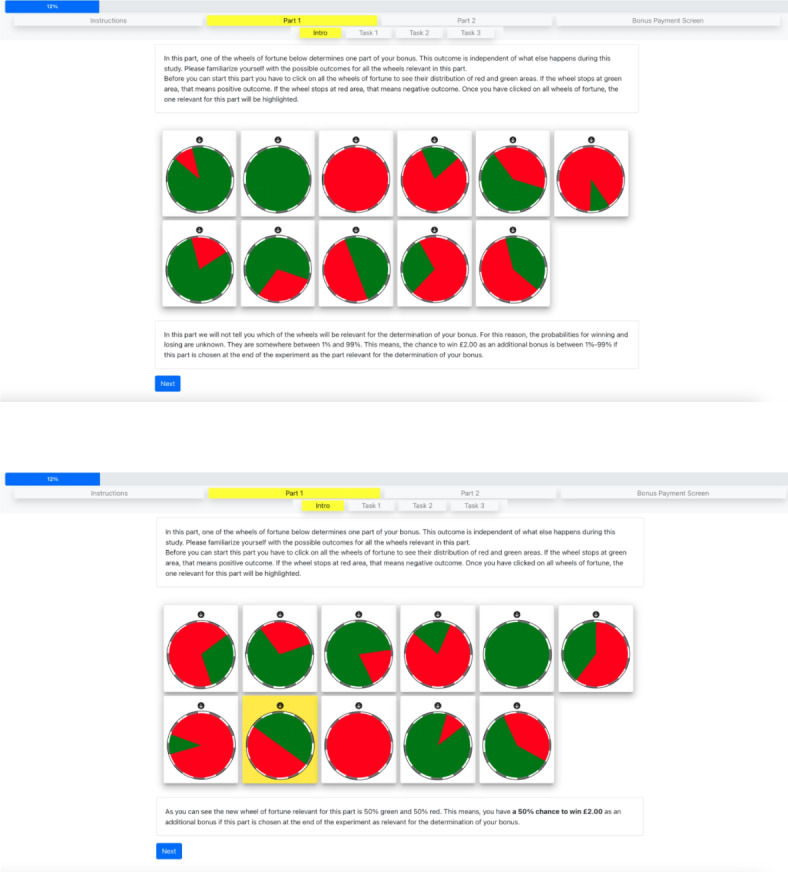

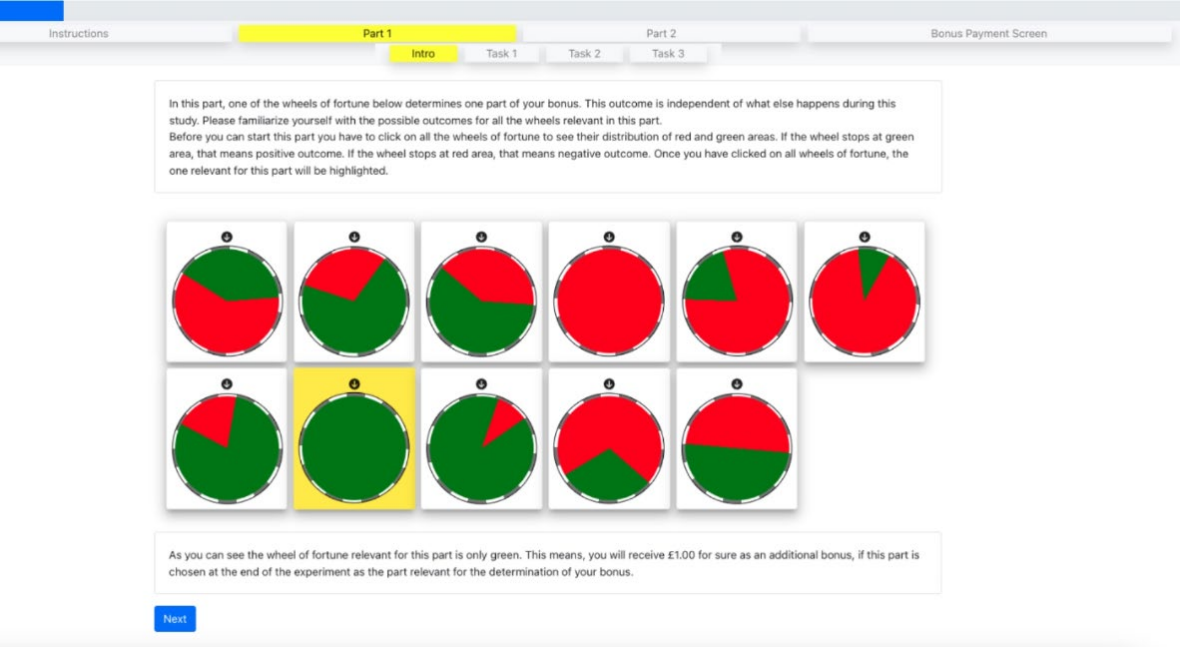
Screenshots of “ambiguity”, “risk” and “control” conditions for Study 4.

Figure 5 shows the screenshots of the conditions, particularly how we operationalized the information about the existence and type of uncertainty using spinning wheels.

## Data Availability

The raw and processed data that support the findings of this study as well as all analysis scripts are available in OSF (project https://osf.io/5qtez/) with the identifier 10.17605/OSF.IO/5QTEZ.

## Code availability

All analysis scripts and supplementary materials are available on the OSF, https://osf.io/5qtez/ with the identifier 10.17605/OSF.IO/5QTEZ.
